# Cardiomyopathy in Genetic Aortic Diseases

**DOI:** 10.3389/fped.2021.682390

**Published:** 2021-07-15

**Authors:** Laura Muiño-Mosquera, Julie De Backer

**Affiliations:** ^1^Department of Pediatrics, Division of Pediatric Cardiology, Ghent University Hospital, Ghent, Belgium; ^2^Center for Medical Genetics, Ghent University Hospital, Ghent, Belgium; ^3^Department of Cardiology, Ghent University Hospital, Ghent, Belgium

**Keywords:** Marfan syndrome, HTAD, cardiomyopathy, arrhythmia, myocardial disease, *FBN1* gene

## Abstract

Genetic aortic diseases are a group of illnesses characterized by aortic aneurysms or dissection in the presence of an underlying genetic defect. They are part of the broader spectrum of heritable thoracic aortic disease, which also includes those cases of aortic aneurysm or dissection with a positive family history but in whom no genetic cause is identified. Aortic disease in these conditions is a major cause of mortality, justifying clinical and scientific emphasis on the aorta. Aortic valve disease and atrioventricular valve abnormalities are known as important additional manifestations that require careful follow-up and management. The archetype of genetic aortic disease is Marfan syndrome, caused by pathogenic variants in the *Fibrillin-1* gene. Given the presence of fibrillin-1 microfibers in the myocardium, myocardial dysfunction and associated arrhythmia are conceivable and have been shown to contribute to morbidity and mortality in patients with Marfan syndrome. In this review, we will discuss data on myocardial disease from human studies as well as insights obtained from the study of mouse models of Marfan syndrome. We will elaborate on the various phenotypic presentations in childhood and in adults and on the topic of arrhythmia. We will also briefly discuss the limited data available on other genetic forms of aortic disease.

## Introduction and Definition of the Diseases: Marfan Syndrome and Heritable Thoracic Aortic Disease

Heritable Thoracic Aortic Diseases (HTAD) encompasses a spectrum of genetic conditions in which aortic disease (aneurysms and dissections) has an underlying genetic trigger or familial occurrence. HTAD is classified as syndromic and non-syndromic. The genetic causes fall into several distinct groups of genes coding for (I.) components of the extracellular matrix (ECM) (*FBN1, COL3A1, LOX*); (II.) components involved in the TGFβ pathway (*TGFBR1 and 2, SMAD2 and 3 and TGFB2 and 3*); and (III.) components of the vascular smooth muscle cell apparatus (*ACTA2, MYLK, MYH11, PRKGA1*) (1). The main clinical entities with their respective genes and clinical features are listed in Table 1. Here, only those genes with a definitive or strong association with HTAD are listed. There are many more candidate genes on the horizon, and this list keeps growing.

**Table 1:** Main clinical features and genes^*^ associated with Heritable Thoracic Aortic Aneurysm and Dissection.

	**Disorder**	**Gene(s)**	**Main cardiovascular features**	**Additional clinical features**
**SYNDROMIC HTAD**				
Extracellular matrix	Marfan Syndrome	*FBN1*	Aortic root aneurysm and dissection Mitral valve prolapse Ventricular dysfunction and arrythmia	Lens luxation Skeletal features
	Vascular Ehlers-Danlos syndrome	*COL3A1*	Aortic and major branching vessel dissection/rupture often without preceding dilatation	Thin, translucent skin Dystrophic scars Facial characteristics Bowel/uterine rupture Club feet Carotido-Cavernous fistulae
TGFβ-pathway	Loeys-Dietz syndrome	*TGFBR*1/2 *TGFβ*2/3 *SMAD*3	Aortic root aneurysm and dissection Arterial aneurysms and dissections Arterial tortuosity Mitral valve prolapse Congenital cardiac malformations	Bifid uvula/cleft palate Hypertelorism Craniosynostosis Pectus abnormalities Scoliosis Club feet Premature Osteoarthritis (*SMAD3*)
VSMC contractile apparatus	Smooth muscle cell dysplasia syndrome	*ACTA2 R189*	Patent ductus arteriosus Aorto-pulmonary window Aortic root dilatation	Congenital bilateral Mydriasis Moya-Moya like cerebral vessel anomalies Gut malrotation
**NON-SYNDROMIC HTAD**				
Extracellular matrix	FTAA	*FBN1* *LOX*	Aortic root aneurysm and dissection BAV (*LOX*)	Variable expression of some systemic features (pectus abnormalities, dural ectasia)
TGFβ-pathway	FTAA	*TGFBR*1/2 *SMAD2/*3 *TGF*β2/3	Thoracic aortic aneurysm and dissection Intracranial aneurysms Mitral valve prolapse	Variable expression of some systemic features
VSMC contractile apparatus	FTAA	*ACTA*2 *MYLK* *PRKG*1 *MYH11*	Cerebrovascular and coronary artery disease (*ACTA 2*) Patent Ductus Arteriosus (*MYH11, ACTA2*)	Livedo reticularis and iris flocculi (*ACTA2*) Gastro-intestinal abnormalities (*MYLK*)

*BAV, bicuspid aortic valve; FTAA, familial thoracic aortic aneurysm; HTAD, heritable thoracic aortic aneurysm and dissection; VSMC, vascular smooth muscle cell*.

* *Only genes with a strong or definitive association are listed*.

The paradigm syndromic HTAD entity is Marfan syndrome (MFS). MFS is an inherited connective tissue disease caused by pathogenic variants in the Fibrillin-1 gene (*FBN1*), which codes for the ECM protein fibrillin-1. The condition was first described in 1895 by the French pediatrician Antoine Bernard Marfan who described a constellation of skeletal abnormalities characterized by joint contractures and conspicuously long fingers (arachnodactyly) in a young girl (2). It took over 50 years for the clinical picture of Marfan syndrome to be more clearly defined in the seminal work of Victor McKusick. He described the condition as a connective tissue disease with cardiovascular involvement. Without knowing the underlying molecular defect, he very accurately reported that “Clinically, Marfan syndrome behaves as an abiotrophy of some connective tissue” (3). By this time, cardiovascular involvement had been consistently reported, along with the skeletal and ocular organ systems' involvement. The concept of an abnormality in elastic fibers in the aorta as a cause for the characteristic aortic aneurysms and dissections was put forward. In addition, involvement of the veins, the heart valves and also the endocardium and myocardium were suspected. The latter fact is of particular value in the context of this review.

Unraveling the structural components of connective tissue again took several decades. Evidence for the link between connective tissue and the clinical entity of Marfan syndrome was first provided by immunohistochemic studies using antibodies for fibrillins, showing deficiencies in the amount and distribution of microfibrillar fibers in skin samples from patients with MFS (4). The identification of pathogenic missense variants in the *FBN1* gene in two patients with Marfan syndrome in 1991 provided final confirmation (5). Fibrillins are large structural macromolecules that contribute to the integrity and function of all connective tissues. According to the initial concept, fibrillin microfibrils mainly served as a scaffold for elastic fiber formation. Biochemical investigations and genetic evidence from both humans and mice have now uncovered many more functions of fibrillin microfibrils. Today, we know that fibrillin microfibrils have essential tissue-specific architectural functions beyond serving as scaffolds for elastin deposition. More recently, an important functional role of fibrillin microfibrils has emerged: fibrillin microfibrils target and sequester members of the TGFβ superfamily of growth factors. In this manner, the structures of fibrillin microfibrils collaborate with biological functions to shape and maintain connective tissues (6). The combined structural and functional role of fibrillins nicely illustrates the current concept of mechanobiology underlying the pathophysiology of cardiovascular disease in MFS. Through interactions between vascular smooth muscle cells (in the aorta) or cardiomyocytes (in the myocardium) and the ECM, the cells can sense changes in mechanical forces of the ECM. These mechanical signals are converted into biochemical or electrical signals, thereby enabling a responsive cellular adaption and remodeling. This process, which is bidirectional, is called mechanobiology. The composition of the ECM and the proportion and the expression of each protein can have a profound influence on cardiac structure and compliance that will determine its hemodynamic functions. One of the major myocardial ECM components is collagen, which will, when present in excessive concentrations lead to myocardial fibrosis and distortion of the myocardial architecture. Fibrosis is prevalent in many acquired cardiac diseases and underlies several adverse cardiac events, such as heart failure, arrhythmia, and death. Increased fibrillin-1 expression has been reported in the context of myocardial fibrosis (7) and gene expression studies have targeted genes involved in the ECM as highly enriched in patients with cardiomyopathy (CMP) with fibrosis and cardiac remodeling (8). Monogenic forms of CMP caused by pathogenic variants in genes encoding for ECM structural components are scarce. Cases of non-compaction CMP caused by *FBN1* pathogenic variants have been reported (9). Further research in this field is highly relevant, not only to identify potential other genes involved but also since several proteins represent candidate therapeutic targets to prevent or reverse fibrosis (10).

A detailed description of the role of the ECM in the myocardium can be found in a recent review by Frangogiannis (11).

The diagnosis of MFS is based on the identification of clinical manifestations, as defined in the revised Ghent nosology (12). The extent, severity and age of onset of clinical manifestations are highly variable, ranging from severe cardiovascular involvement at birth in the neonatal form to patients developing manifestations only in mid-life. The estimated prevalence of Marfan syndrome is 1 in 3.000–5.000 individuals, with no ethnic or sex predilection (13). Prognosis is mainly determined by progressive dilation of the aorta, leading to aortic dissection and death at a young age. Mean survival of untreated patients is about 40 years. Fortunately, improved management and ongoing research have led to a significant increase in life expectancy of at least 30 years (14, 15) which does not imply that life expectancy in MFS is normal. A recent population study demonstrated a median age at death in MFS patients of 50 years, which is 8–13 years lower than in the general population (16). A critical factor in improving prognosis is the early identification of patients with Marfan syndrome. Precipitating factors reported to accelerate progressive dilatation or dissection include elevated blood pressure, intense physical exercise and pregnancy (17, 18).

## Cardiomyopathy and Arrhythmia in Marfan Syndrome

When referring to Marfan syndrome CMP, two different clinical entities should be distinguished: (1) Heart failure in very young children with MFS (neonates and infants) (2) CMP in classical MFS. We will discuss both entities separately in the next sections.

### Cardiomyopathy in Neonatal and Infantile Marfan Syndrome

**Figure 1:**
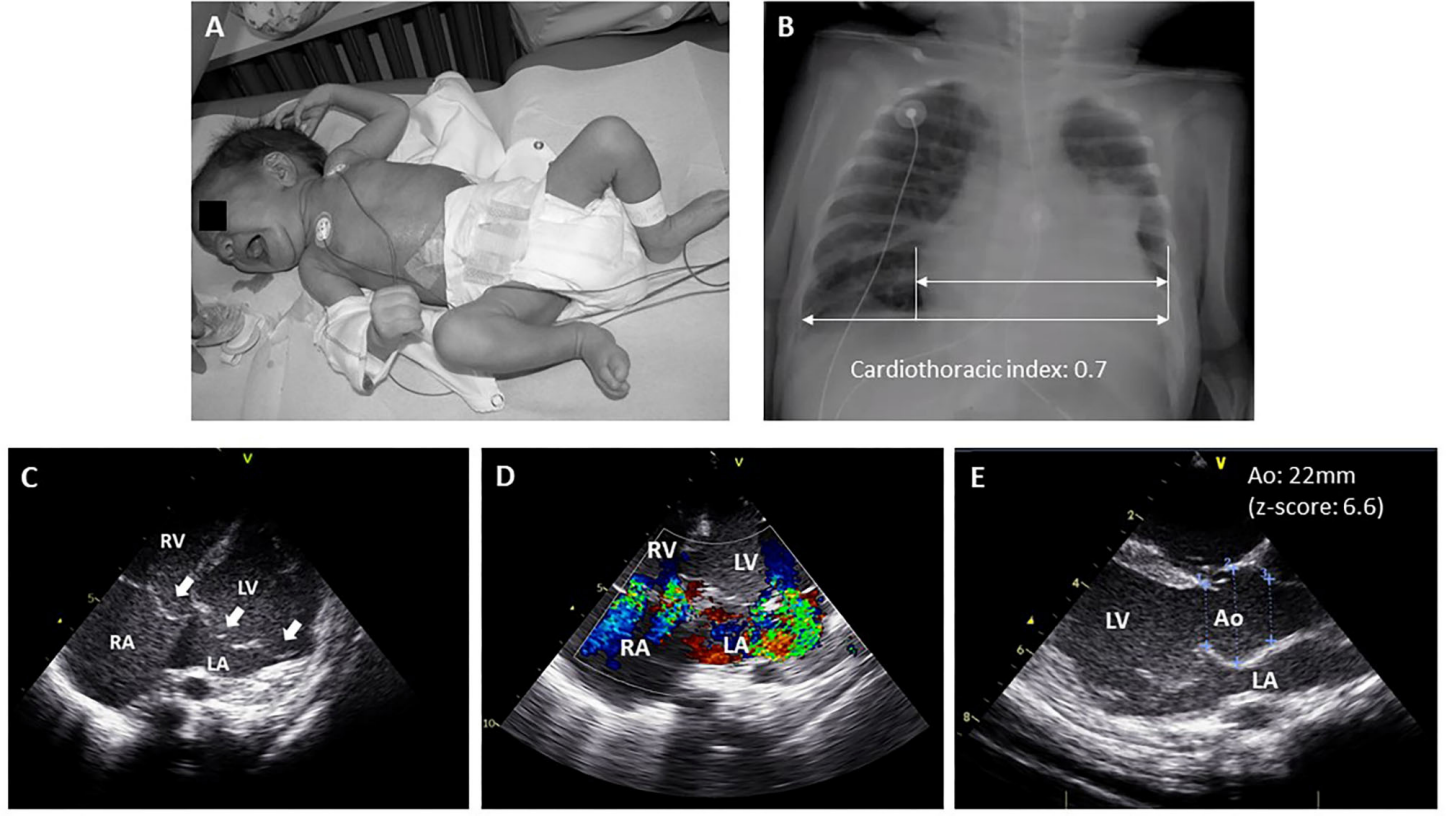
Typical manifestations of neonatal Marfan syndrome. **(A)** Child with neonatal MFS showing arachnodactyly, long feet, crumpled ears, lipodystrophy and mild pectus excavatum. **(B)** Chest X-ray showing cardiomegaly and mild scoliosis. **(C)** Echocardiographic four-chamber view showing mitral and tricuspid valve prolapse (white arrows). **(D)** Echocardiographic apical four-chamber color doppler view showing moderate-severe mitral and tricuspid valve regurgitation. **(E)** Echocardiographic parasternal long axis view showing enlargement of the sinus of Valsalva. Ao, aorta; LA, left atrium; LV, left ventricle; RV, right ventricle; RA, right atrium.

Neonatal MFS (nMFS) is a term usually reserved for very early clinical presentations of MFS even though some patients may present after the 1^st^ month of life (19). The exact prevalence of nMFS is unknown but is much lower than the prevalence of classical Marfan syndrome. The majority of these patients (90–95%) carry a de novo variant in the so-called “neonatal region” (exons 24–32) with a cluster of variants in exons 25–26 (20). Children with nMFS have a typical appearance with dolichocephaly, progeroid appearance, arachnodactyly, crumpled ears, joint contractures and pectus abnormalities (Figure 1A). Some children will exhibit congenital lung emphysema and ocular abnormalities. Unlike classical MFS, the most prominent cardiac problem in children with nMFS is tricuspid and mitral valve prolapse, usually with severe progressive regurgitation leading to congestive heart failure (Figures 1B–E) (20–22). Aortic root dilatation is also commonly present in children with nMFS but does not account for the most significant morbidity and mortality in this age group. Most children with nMFS die within the 1^st^ year of life of cardiac failure (20), although the number of survivors into teenage years is increasing thanks to improvement in care (23, 24).

Children with the infantile form of MFS usually present a less severe course (25). These children might be diagnosed at a very young age (even during the first months of life), and the phenotype is similar to that of nMFS. However, from a cardiovascular perspective, the infantile form of MFS resembles a more severe subform of classical Marfan syndrome. They show severe aortic root dilation at a young age, but atrioventricular valve dysfunction is typically less prominent than in the neonatal form (21, 26). These children usually present left ventricular dilatation, even with mild valvular regurgitation, but preserved left ventricular function.

### Cardiomyopathy in Classic Marfan Syndrome

For obvious reasons, cardiovascular research and management recommendations for classic MFS have heavily focused on aortic disease. Interest in studying myocardial involvement was raised in the nineties with a study from Savolainen and colleagues, indicating abnormal diastolic function in children with MFS, assessed by cardiac magnetic resonance (CMR) (27).

Moreover, several (historical) series on survival in MFS have listed heart failure as one of the leading causes of death. Estimates vary between 5 and 30% (16, 28) putting heart failure at least at an equal level as aortic dissection. End-stage heart failure necessitating heart transplantation in patients with MFS has been reported in several case reports and small series (29–31). In most of these patients, heart failure was triggered by underlying severe valvular disease (aortic and/or mitral valve regurgitation).

In addition to these extrinsic (secondary) causes of heart failure, several reports from independent researchers have shown intrinsic myocardial dysfunction in MFS. The reported prevalence of what is now known as Marfan CMP ranges from 3% (32) to 68% (33) across different series, depending on the definition and population characteristics. Involvement of both left and right ventricles with systolic and diastolic dysfunction has been reported (27, 34–39) (Table 2). Although myocardial dysfunction is mostly mild and subclinical and does not progress much over time, some patients will present in overt heart failure. A possible link between intrinsic CMP and an unfavorable course in the event of an additional hemodynamic trigger such as valvular dysfunction and/or aortic root replacement has not yet been demonstrated but seems plausible. Whether these hemodynamic changes trigger myocardial fibrosis, as seen in other types of CMP (50), is also not clear in MFS. A small study in children with MFS and Loeys-Dietz syndrome (LDS) showed increased left- and right ventricular volumes and diffuse myocardial fibrosis on CMR in comparison to healthy control subjects (51). Further evidence is necessary to elucidate whether diffuse fibrosis is present in MFS and influences clinical outcome. Whether the type of underlying pathogenic *FBN1* variant plays a role in defining the risk for developing CMP is still unclear. Two independent studies observed a higher incidence of left ventricular dilation and decreased left ventricular function in patients carrying non-missense variants (46, 49), which is in line with recent data on genotype-phenotype correlations in patients with Marfan syndrome, indicating a worse cardiovascular phenotype in patients harboring non-missense variants predicted to result in a haploinsufficient effect (52).

**Table 2:** Overview of the studies assessing cardiac function in Marfan syndrome.

**AuthorYear**	**Subjects (mean age/range)**	**Method**	**Main findings**
**CARDIOMYOPATHY**			
Savolainen (27) 1994	22 MFS (3-14.5 yr) 22 control	Echocardiography Cardiac MRI	Similar LV diameter and systolic function LV relaxation impairment in MFS
Porciani (40)2002	20 MFS (29.5 yr) 8 MASS 28 controls	Echocardiography	Similar LV diameter and systolic function LV diastolic dysfunction in MFS
Chatrath (41)2003	36 MFS without valvular disease	Echocardiography	19% LV dilatation Normal systolic function
Meijboom (34)2005	234 MFS (29 yr)	Echocardiography	Normal systolic function and ventricular dimensions in most of the patients. Mild involvement in a subgroup
De Backer (36)2006	26 MFS (32 yr) 26 controls	Echocardiography Cardiac MRI	Mild but significant impairment of LV systolic and diastolic dysfunction in MFS
Das (35)2006	40 MFS (17 yr) 40 controls	Echocardiography	Impaired relaxation independent of aortic root dilation
Rybczynski (37)2007	55 MFS 86 controls	Echocardiography	Reduced systolic and early diastolic tissue doppler velocities in adults with MFS
Kiotsekoglou (38)2008	66 MFS (15-58 yr) 61 controls	Echocardiography	LV systolic dysfunction is significantly reduced in MFS
Kiotsekoglou (42)2008	72 MFS (32 yr) 73 controls	Echocardiography	Significant biventricular diastolic and biatrial systolic and diastolic dysfunction in MFS patients
Kiotsekoglou (43)2009	66 MFS (15-58 yr) 61 controls	Echocardiography	Primary impairment of RV systolic function in MFS
Alpendurada (39)2010	68 MFS (33.9 yr)	Cardiac MRI	Primary cardiomyopathy in a subgroup of MFS patients
de Witte (44)2011	144 MFS 19 controls	Cardiac MRI	Lower RV- and LVEF 9% LVEF <45% Result independent of aortic elasticity of β-blocker use
Scherpetong (45)2011	50 MFS (35.2 yr) 50 controls	Echocardiography Longitudinal, FU: 4 yr	Lower RV and LV strain rate in MFS No progression during FU in the majority.
Aalberts (46)2014	183 MFS (33.5 yr)	Echocardiography	LV dilatation is more frequent in patients with a non-missense *FBN1* pathogenic variant
Campens (47)2015	19 MFS (adults)	Echocardiography Longitudinal, FU: 6 yr	No further echocardiographic deterioration of LV function during FU
Gehle (48)2016	217 MFS (30 yr)	Echocardiography	Increased Nt-ProBNP levels Increased LV diameters LV diastolic dysfunction
Muiño-Mosquera (49)2020	86 MFS (36.3 yr) 40 controls	Echocardiography Nt-ProBNP	Increased Nt-ProBNP levels, increased LV diameters and decreased RV function Patients after aortic surgery of with valvular disease more affected

*FU, Follow-up; LV, Left ventricle; LVEF, Left ventricle ejection fraction; MASS, mitral valve, aortic, skin, skeletal features; MFS, Marfan syndrome; MRI, Magnetic Resonance Imaging; Nt-ProBNP, N-terminal pro-hormone brain natriuretic peptide; RV, Right ventricle; yr, years*.

### Arrhythmia in Marfan Syndrome

**Figure 2:**
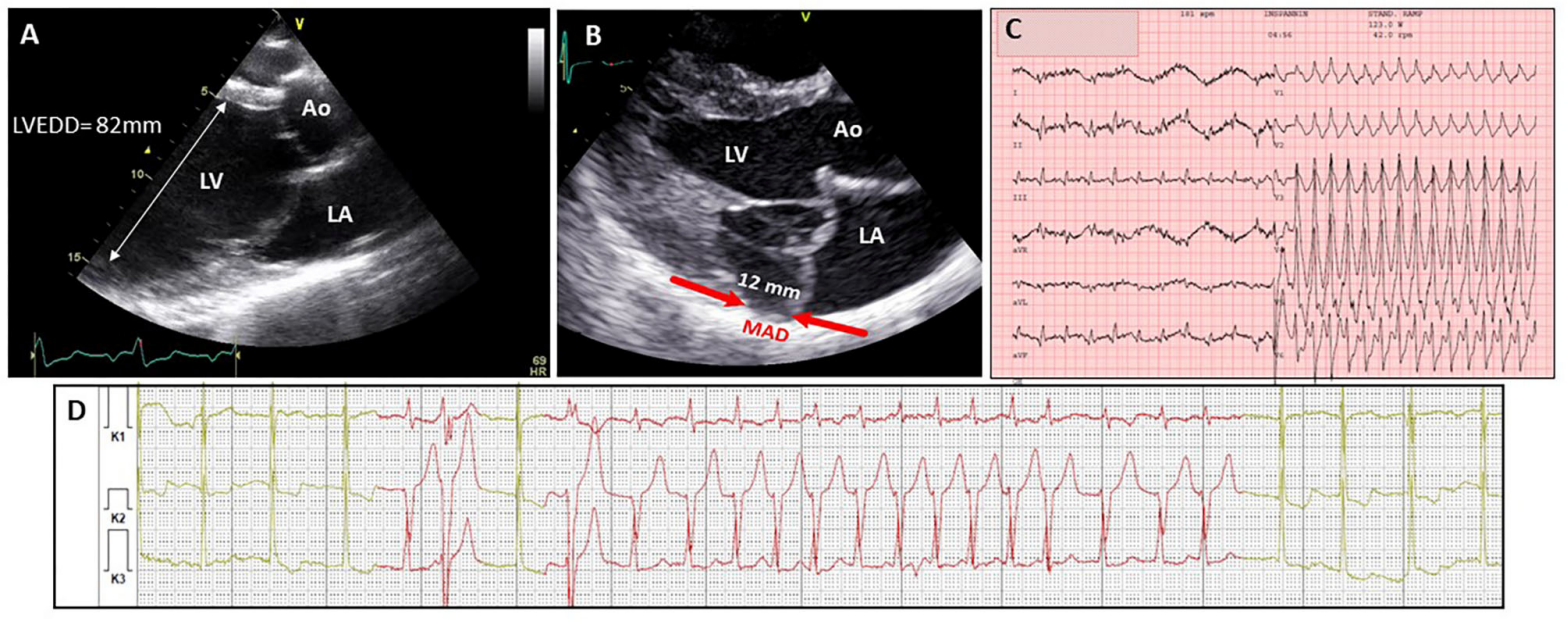
Different manifestations of myocardial disease in classical Marfan syndrome. **(A)** Echocardiographic image (2D TTE PSLAX view) in a 16 yr old male showing severe dilatation of the left ventricle. **(B)** Echocardiographic image (2D TTE PSLAX view) showing mitral annular disjunction. **(C)** ECG recordings in 28 yr old male showing ventricular tachycardia followed by presyncope during exercise test 6 m after mitral valve surgery and aortic root replacement **(D)** ambulatory electrocardiogram recording in a 57 yr old female with frequent episodes of non-sustained ventricular tachycardia. Ao, aorta; LA, left atrium; LV, left ventricle; LVEDD, left ventricular end-diastolic diameter; MAD, mitral annular disjunction.

Next to impaired function, arrhythmia may be considered as another manifestation of myocardial disease in MFS (Figure 2). Most of the evidence for arrhythmia in MFS comes from the study of adult cohorts, in which children were only occasionally included (Table 3). In these studies, significant ventricular ectopy (defined as >10 premature ventricular contractions per hour) was found in 20–35% of the patients (33, 49, 56). A slightly lower percentage of patients with MFS (10–25%) also present non-sustained ventricular tachycardia (NSVT) on 24h ambulatory monitoring (33, 49, 54, 56). Ventricular tachycardia (VT) and sudden cardiac death (SCD) have respectively been reported in 7–9 and 4% of the patients (33, 55, 56, 58).

**Table 3:** Overview of the published papers evaluating ventricular arrhythmia in MFS.

**AuthorYear**	**Subjects (mean age/range)**	**Method**	**Main findings**
**VENTRICULAR ARRHYTHMIA**			
Chen (53)1985	24 MFS (children)	Echocardiography AECG	Serious ventricular dysrhythmia can occur in children with MFS with or without valve disease. The dysrhythmia appears to progress with age
Savolainen (54)1997	45 MFS (34 yr) 45 controls	AECG	Patients with MFS have a higher prevalence of cardiac dysrhythmias than healthy persons
Yetman (33)2003	70 MFS (0–52 yr)	Echocardiography ECG AECG FU: 24 yr	Sudden death occurring in 4% of MFS patients LV dilation may predispose to alterations of repolarization and fatal ventricular arrhythmias
Hoffman (55)2013	77 MFS (36.1 yr)	Echocardiography ECG AECG NT-ProBNP	NT-ProBNP predicts adverse arrhythmogenic events in patients with MFS
Aydin (56)2013	80 MFS (42 yr)	Echocardiography ECG AECG NT-ProBNP	MFS is associated with an increased risk for arrhythmia. Risk factors: Ventricular arrhythmia on ECG, signs of myocardial dysfunction and pathogenic variants in exons 24–32
Mah (57)2018	274 MFS (10.8 yr)	Echocardiography AECG	VE and supraventricular ectopy is rare in children with MFS. Increased LV diameter is related to ventricular ectopy
Muiño-Mosquera (49)2020	86 MFS (36.3 yr) 40 controls	Echocardiography ECG AECG Nt-ProBNP	VE and NSVT were more frequent in MFS than in age- and sex-matched controls NSVT was independently associated with increased LV diameter and VES.

*AECG, ambulatory electrocardiogram; ECG, electrocardiogram; FU, Follow-up; LV, Left ventricle; MFS, Marfan syndrome; NSVT, non-sustained ventricular tachycardia; VE, ventricular ectopy; yr, years*.

One of the first studies of arrhythmia in children with MFS took place in the early 80s (53). In this study, eight of the 24 children with MFS (33.3%) presented with ventricular arrhythmia, three of whom showed ventricular tachycardia. Ventricular arrhythmia was associated with mitral valve prolapse and prolonged repolarization time. Another interesting early observation comes from the first (and only) randomized trial assessing the effect of propranolol on aortic root dilatation in patients with Marfan syndrome (59). Two deaths were observed in the control group of this trial, one 14 year old boy and one 18 year old women, both of which had mitral-valve prolapse and a history of paroxysmal tachyarrhythmia. Aortic dissection was excluded in both postmortem. While this study lacks power to draw meaningful conclusions from this observation, the history of arrhythmia is remarkable and one may even hypothesize that propranolol had a protective effect in the treatment group. A more recent study, an ancillary analysis of the Pediatric Heart Network (PHN) Marfan trial (57) also studied arrhythmia in children with MFS. The primary aim of the PHN Marfan trial was to compare aortic outcome in 608 children with MFS, randomly assigned to treatment with atenolol or losartan. As part of this study, a subgroup of patients (*n* = 274) underwent 24h ambulatory monitoring. Ventricular ectopy was present in 7% of these children, but (NS)VT was not observed. The prevalence of ventricular arrhythmia in these two studies is clearly different but probably reflects clinical variability among the cohorts. On the contrary, these studies show that ventricular arrhythmia, although uncommon, can present at an early age. Physicians taking care of children with MFS should also be vigilant for these complications.

The mechanisms underlying severe ventricular arrhythmia in MFS are not clear yet. Judging from the numbers above, it seems that age might play an important factor, although this has not been clearly shown (49). So far, an enlarged LV diameter appears to be the most consistent independent factor associated with an arrhythmic event (33, 49, 55). Other factors like mitral valve prolapse, mitral valve regurgitation and previous aortic surgery have also been associated with ventricular arrhythmia in a variable amount of studies (33, 49, 56, 60). Two studies from the Hamburg Marfan center indicated that NT-proBNP level is the strongest independent predictor of arrhythmogenic events (55, 56). In our own study, NT-pro-BNP levels were also elevated in patients presenting NSVT, although not significantly (49).

Lately, mitral annular disjunction (MAD), defined as the separation between the posterior mitral valve leaflet hinge point and the left ventricular myocardium, has gained interest in patients with MFS. A recent study shows that 46% of patients have MAD and that presence of MAD is associated with a worse aortic and mitral outcome (61). In non-MFS subjects, MAD has also received particular attention as a potential marker or substrate of ventricular arrhythmia and SCD (62). In our recent study in 142 patients with MFS, MAD was present in 36% of the cohort and was associated with the presence of VT and SCD (manuscript in press, JAMA Cardiology). Ventricular ectopy in patients with mitral valve prolapse and MAD is presumed to be partially due to regional stretch leading to fibrosis of the papillary muscles (63). Whether the same underlying mechanism is present in MFS is not clear yet and deserves further study.

Besides an abnormal substrate, triggering factors might also play a role in developing arrhythmia in MFS. Subtle ECG changes have been identified in patients with MFS independent of aortic root diameter, mitral and/or tricuspid valve prolapse or chamber dimension and function. Patients with MFS display slightly prolonged PQ- and QTc-intervals compared to healthy controls (49, 54).

Atrial arrhythmia in MFS has been given less attention. Atrial fibrillation seems to be more common in MFS than in the general population and seems to occur at a younger age (49, 64). Other types of atrial arrhythmias have been described in MFS, mainly re-entry tachycardia, but it does not seem more frequent in MFS than in the general population (57).

## The Myocardium in (Marfan) Mouse Models

The presence of fibrillin-1 in the myocardium has clearly been evidenced in wild-type mice. Histology shows more abundant amounts in the atria compared to the ventricles and a distinct spatial arrangement in the ventricular myocardium with more fibrillin-1 in the inner trabeculated part when compared to the outer trabeculated part (65). The inner myocardium is more prone to shearing forces during ventricular contraction, and connective tissue aligning these lamellae play a role in providing mechanical coupling and preventing overextension (66). Based on the known elastic properties of fibrillin-1 and its observed spatial arrangement at the level of the inner myocardium, it is conceivable that fibrillin-1 provides the required elasticity to the myocardial tissue allowing shearing of the muscle lamellae. This hypothesis is supported by the limited presence of elastic fibers in the myocardium, making fibrillin-1 fibers the most important myocardial ECM component with elastic properties (67). In addition, the role of fibrillin-1 in providing elasticity to the myocardium is also observed in the *fbn1*^*mgR*/*mgR*^ Marfan mouse model. A decrease in passive filling properties of the left ventricle in this model suggests an impaired elastic recoil of the left ventricle (68). It is assumed that the underlying abnormality in the *FBN1* gene in MFS results in an impaired signaling function of fibrillin microfibrils in the ECM and that mechanical factors such as volume- or pressure overload are not correctly compensated which in turn leads to myocardial dysfunction. An example of pressure overload is provided by Rouf and colleagues who observed CMP after partial ligation of the aortic arch in *fbn1*^*C*1039*G*/+^ mice (69). Volume overload caused by valvular regurgitation (both mitral and aortic valve) results in dilated CMP in the same mouse model (70).

These observations support the concept of mechanobiology as a possible cause for CMP (71). As already mentioned, the model of abnormal mechanobiology has also been introduced in recent years to explain aortic pathology in MFS (72).

In addition to the evidence for functional impairment of the myocardium in mouse models for MFS, some interesting morphological alterations are also worth mentioning. In the *fbn1*^*mgR*/*mgR*^ mice, an age-dependent decrease in myocardial compaction was noted on routine staining compared to WT mice sections (65). As mentioned above, fibrillin-1 fibers align the periphery of inner myocardial muscle lamellae in WT tissue. It appears that fibrillin-1 functions as a glue in the trabeculated myocardium, strengthening intercellular connections through cell-ECM-cell interactions by forming molecular bridges between the pericellular and interstitial ECM. Abnormal fibrillin-1 fibers may lead to loosened connections or non-compaction. Interestingly, left ventricular non-compaction has also been linked to pathogenic *FBN1* variants in humans (9, 73). Next to the loss of myocardial compaction, macroscopic inspection of the right ventricle free wall in the *fbn1*^*mgR*/*mgR*^ mouse model revealed the presence of (multiple) pseudoaneurysms. In WT mice, fibrillin-1 fibers cross the entire right ventricle free wall from lumen to pericardium. In the setting of MFS, reduced amounts of fibrillin-1 fibers may result in the formation of a gap crossing the entire right ventricle free wall. To our knowledge, there is no confirmation of such findings in human Marfan disease, but this definitely deserves further research.

## Recommendation for Follow-Up and Treatment

Myocardial disease is an upcoming problem in MFS, especially in adults. As indicated above, a subgroup of children is also at risk. Careful monitoring of myocardial function and potential complications such as arrhythmia in patients with MFS is warranted. Yearly echocardiographic evaluation and follow-up should include assessment of biventricular systolic and diastolic function. Whether CMR will aid in risk stratification in patients with MFS still needs to be elucidated. Patients with enlarged left ventricular diameter, patients with MAD and patients with palpitations, (pre)syncope, or chest pain can benefit from an ambulatory electrocardiogram. As in general recommendations NT-pro-BNP levels are useful to monitor heart failure and may be useful for risk stratification for arrhythmia in Marfan syndrome (49, 55, 56).

There is currently no evidence that treatment of arrhythmia and heart failure in patients with MFS should differ from the guidelines for other non-MFS patients. If congestive heart failure is present as a result of valvular dysfunction, afterload-reducing agents can improve cardiovascular function. Whether losartan (or other angiotensin receptor blockers), drugs known for their beneficial effect in heart failure should be considered a first line choice in these patients is not known. In a small study with losartan in patients with MFS, we did not observe a significant effect on LV dimensions- and function (74). Indications for surgical intervention for valvular disease should follow the general guidelines (75).

End-stage heart failure is uncommon in patients with MFS but heart transplantation may be considered. Although early results with this procedure in MFS were not good (30), subsequent reports were more encouraging (29). A major concern relates to complications occurring in the distal aorta, which are mostly (but not exclusively) occurring in patients with pre-existing aortic complications (31, 76). Given the inherent fragility of the aortic tissue, the use of assist devices should be limited, although successful cases have been reported (77).

The role of β-blocker therapy for the treatment of arrhythmia or prevention of SCD in MFS is not clear yet. In one study, two of the three patients presenting SCD were under treatment with β-blocker (33). In our study, one patient presenting VT showed progressive ventricular ectopy under β-blocker treatment and episodes of sustained VT were only controlled after treatment with amiodarone (49). In the absence of specific risk factors to stratify patients at risk of VT or SCD, indications for implantable defibrillator should follow the general guidelines (78).

## Evidence of Cardiomyopathy and Arrhythmia in Other HTAD

There is very scarce literature on myocardial disease and arrhythmia in HTADs and evidence of myocardial dysfunction in this group of diseases, mainly comes from case reports and casual description from registries.

LDS was first described in 2005 as a connective tissue disorder with important vascular involvement (79). Shortly thereafter, a case report describing a patient carrying a pathogenic variant in *TGFBR1* with heart failure necessitating heart transplant was published (80). Several other case reports mentioned both heart failure and SCD in patients carrying variants in other genes: *TGFB2, TGFBR2* and *SMAD3* (81–84). Myocardial disease with left ventricular hypertrophy in 16% and VE in 19% of the patients carrying variants in *SMAD3* was already reported in one of the first case series characterizing patients with pathogenic variants this gene (85). Only one systematic study evaluating repolarization abnormalities in patients carrying variants in *TGFBR2* has been published (86). In this study, two patients presented SCD and 47% of patients presented abnormal repolarization characterized by slight prolongation of the QTc interval, abnormal ST-segment and abnormal T-U wave.

## Conclusion

Findings based on human studies and from mouse models provide increasing evidence for the clinical relevance of CMP in genetic aortic disease, but more data are required for further confirmation and delineation.

Careful monitoring of myocardial function and potential consequences such as arrhythmia in patients with MFS and other HTAD is warranted. Further study to understand the underlying pathophysiology of myocardial disease is necessary to identify better treatment targets and improve patient's outcome.

## Author Contributions

JDB and LM-M wrote and reviewed this paper. Both authors contributed to the article and approved the submitted version.

## Conflict of Interest

The authors declare that the research was conducted in the absence of any commercial or financial relationships that could be construed as a potential conflict of interest. The reviewer SM declared a past co-authorship with one of the authors JDB.
